# Pseudoprogression of cutaneous squamous cell carcinoma invading the super vermillion border following programmed death‐1 inhibitor monotherapy

**DOI:** 10.1002/ski2.400

**Published:** 2024-05-30

**Authors:** Sach Thakker, Naghmeh Yousefzadeh, Jafar Al‐Mondhiry

**Affiliations:** ^1^ Georgetown University School of Medicine Washington District of Columbia USA; ^2^ Sonic Healthcare Dermatopathology Sterling Virginia USA; ^3^ Inova Schar Cancer Institute Fairfax Virginia USA; ^4^ University of Virginia School of Medicine Charlottesville Virginia USA

## Abstract

A 55‐year‐old woman with a moderately differentiated cutaneous squamous cell carcinoma (cSCC) of the upper lip experienced initial tumour growth and new lymphadenopathy after starting immunotherapy with Cemiplimab, but achieved complete remission with no adverse events after five infusions. This case underscores the potential of immunotherapy for cSCC in sensitive head and neck areas and illustrates the phenomenon of pseudoprogression, where apparent tumour growth can occur before clinical improvement.

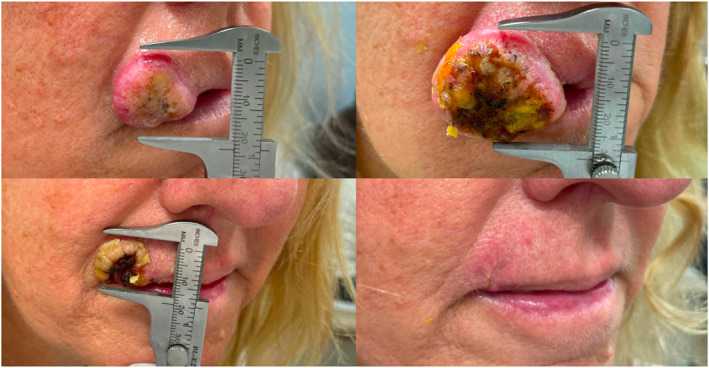

A 55‐year‐old Caucasian female presented for evaluation of a tumour of the right upper lip (2.3 × 2.5 cm) (Figure 1a). Shave biopsy confirmed moderately differentiated cutaneous squamous cell carcinoma (cSCC), keratoacanthoma subtype, without perineural invasion (Figure 2). Cemiplimab 350 mg was administered intravenously every 3 weeks. One week after the first infusion, clinical exam demonstrated substantial growth in the tumour (3 × 3.5 cm) in addition to new cervical lymphadenopathy (Figure 1b). US‐guided lymph node biopsy showed reactive hyperplasia with no tumour involvement. Clinical regression of the tumour was noted following second infusion, 3 weeks after the first infusion (Figure 1c). With continued treatment, her lymphadenopathy resolved, and rapid clinical resolution of the tumour was observed without any immune‐related adverse events (Figure 1d). Due to complete clinical remission, treatment was discontinued after the fifth infusion (13 weeks after first infusion). She remains clinically and radiographically disease free at the 1 year follow up.

**FIGURE 1:**
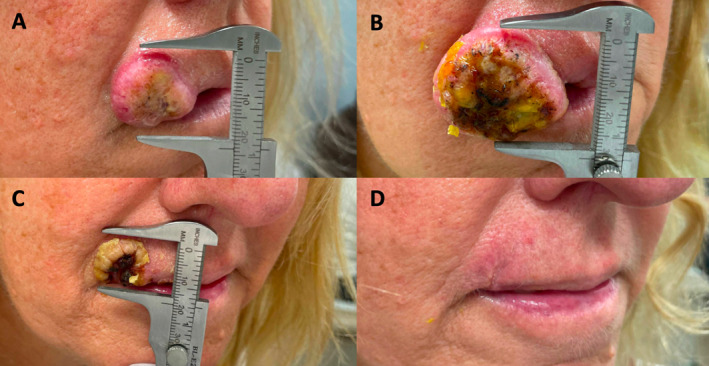
(a) Initial presentation of cutaneous squamous cell carcinoma of the right upper lip. (b) Pseudoprogression of tumour following first infusion of cemiplimab. (c) Clinical regression of tumour following second cemiplimab infusion. (d) Complete response of tumour following fourth cemiplimab infusion.

**FIGURE 2:**
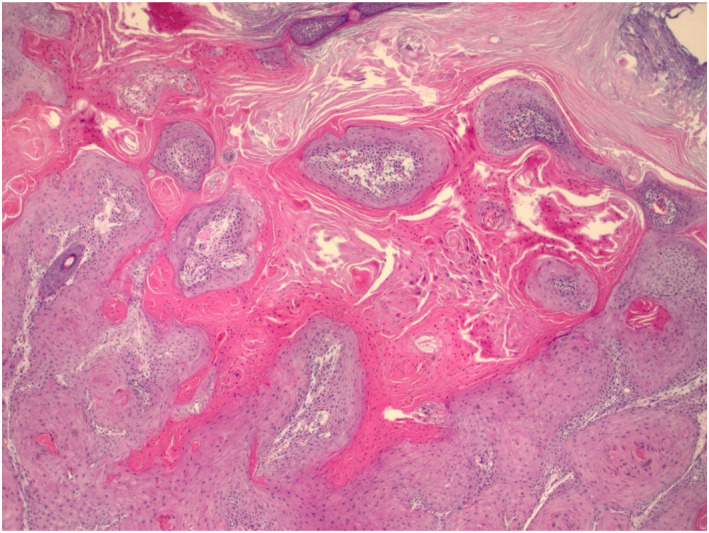
Histologic stain: sections show an endophytic proliferation of atypical squamous cells with abundant pale, eosinophilic cytoplasm. The neoplasm forms a cup‐shaped depression filled with abundant keratin, surrounded by irregular lobules of squamous cells. Nuclear pleomorphism, dyskeratosis, and mitotic figures are seen within the atypical cells.

While definitive radiation or surgery is the standard of care for cSCC, immunotherapy should be considered for tumours affecting sensitive portions of the head and neck, based on growing literature showing high sensitivity of cSCC to programmed death‐1 inhibition with a limited toxicity profile.^1^, ^2^ Furthermore, this case highlights a striking clinical presentation of pseudoprogression, which can be seen with even large increases in tumour volume (in this case, 50%) and new potential disease sites. Psuedoprogression of head and neck cSCC is a rare occurrence, with one cohort study reporting an incidence of 1.3%.^3^

## CONFLICT OF INTEREST STATEMENT

None to declare.

## AUTHOR CONTRIBUTIONS

**Sach Thakker**: Conceptualization (lead); writing – original draft (lead); writing – review & editing (equal). **Naghmeh Yousefzadeh**: Investigation (supporting); writing – original draft (supporting). **Jafar Al‐Mondhiry**: Conceptualization (equal); supervision (equal); writing – original draft (equal); writing – review & editing (equal).

## ETHICS STATEMENT

Not applicable.

## PATIENT CONSENT

Consent for the publication of all patient photographs and medical information was provided by the authors at the time of article submission to the journal, stating that all patients gave consent for their photographs and medical information to be published in print and online and with the understanding that this information may be publicly available.

## Data Availability

Data sharing not applicable to this article as no datasets were generated or analysed during the current study.
